# Validation of the mSOAR and SOAR scores to predict early mortality in Chinese acute stroke patients

**DOI:** 10.1371/journal.pone.0180444

**Published:** 2017-07-06

**Authors:** Hui Wang, Yuesong Pan, Xia Meng, Chunjuan Wang, Xiaoling Liao, David Wang, Xingquan Zhao, Liping Liu, Hao Li, Yilong Wang, Yongjun Wang

**Affiliations:** 1 Department of Neurology, Beijing Tiantan Hospital, Capital Medical University, Beijing, China; 2 China National Clinical Research Center for Neurological Diseases, Beijing, China; 3 Center of Stroke, Beijing Institute for Brain Disorders, Beijing, China; 4 Beijing Key Laboratory of Translational Medicine for Cerebrovascular Disease, Beijing, China; 5 Monogenic Disease Research Center for Neurological Disorders, Beijing Tiantan Hospital, Capital Medical University, Beijing, China; 6 Department of Epidemiology and Health Statistics, School of Public Health, Capital Medical University, Beijing, China; 7 Illinois Neurological Institute Stroke Network, Sisters of the Third Order of St Francis Healthcare System, University of Illinois College of Medicine, Peoria, IL, United States of America; Massachusetts General Hospital, UNITED STATES

## Abstract

**Background:**

It is unclear in Chinese patients with acute stroke how the SOAR (stroke subtype, Oxfordshire Community Stroke Project classification, age, and prestrike modified Rankin) and mSOAR (modified-SOAR) scores performed in predicting discharge mortality and 3-month mortality. We aimed to validate the predictability of these scores in this cohort.

**Methods:**

Data from the China National Stroke Registry (CNSR) study was used to perform the mSOAR and SOAR scores for predicting the discharge and 3-month mortality in acute stroke patients.

**Results:**

A total of 11073 acute stroke patients were included in present study. The increased mSOAR and SOAR scores were closely related to higher death risk in acute stroke patients. For discharge mortality, the area under the receiver-operator curve (AUC) of the mSOAR and SOAR scores were 0.784 (95% CI 0.761–0.807) and 0.722 (95% CI: 0.698–0.746). For 3-month mortality, they were 0.787 (95% CI: 0.771–0.803) and 0.704 (95% CI: 0.687–0.721), respectively. The mSOAR and SOAR scores showed significant correlation between the predicted and observed probabilities of discharge mortality (mSOAR: *r* = 0.945, *P* = 0.001; SOAR: *r* = 0.994, *P*<0.001) and 3-month mortality (mSOAR: *r* = 0.984, *P*<0.001; SOAR: *r* = 0.999; *P*<0.001).

**Conclusions:**

The mSOAR score predicted reliably the risk of death in Chinese acute stroke patients.

## Introduction

Stroke has been the second leading cause of death and acquired adult disability worldwide [1]. Therefore, the mortality of acute stroke patients is an important endpoint in clinical practice [2–4]. It closely related with agedness, higher neurological severity score and symptomatic intracranial hemorrhage (sICH) [5, 6]. The models were used for predicting the mortality and clinical benefit, which could help clinicians to decide the individual treatments and to inform patients and relatives. In fact, several models have been used to predict clinical functional outcome and risk of sICH in acute stroke patients [7–9], especially the factors associated with stroke mortality have been explored various prognostic models. However, they usually included multiple variables, such as plasma glucose levels and cerebral hematoma volume, which varied with time and not easily obtained. Therefore, a simple and reliable clinical prognostic tool to predict stroke mortality was useful.

The stroke subtype, Oxfordshire Community Stroke Project classification, age, and prestrike modified Rankin (SOAR) score was initially established and validated to predict early mortality in British acute stroke patients [10, 11]. Recently, the modified-SOAR (mSOAR) score were established by adding an initial stroke severity scale (NIHSS), which significantly improved prognostic accuracy of the SOAR score for predicting mortality in acute stroke patients [12]. However, these two models were developed only from the British acute stroke patient population. The prognosis may be different in other ethnic cohort [13]. Thus it is necessary to examine the performance of mSOAR and SOAR score to predict mortality in Asian population.

In this study, we aimed to evaluate the performance of the mSOAR and SOAR score in predicting the short-term and long-term mortality in Chinese acute stroke patients.

## Methods

### Study participants and data collection

Data were derived from the China National Stroke Registry (CNSR). As described in our previous study [14], it was a multicenter, nationwide, well-designed and prospective registry of consecutive acute stroke patients. Briefly, this registry included 21,902 patients who were diagnosed as stroke, intracerebral hemorrhage or transient ischemic attack from Chinese 132 hospitals. The data of demographics, clinical characteristics and outcomes at the 3 months follow-up visits was collected through medical records and telephone interview by trained research coordinators. The telephone follow-up was centralized for all included patients and based on a shared standardized interview protocol. The study protocol was approved by the Ethics Committee of Beijing Tiantan Hospital. Informed consent for participating in the registry was obtained from all patients or their designated relatives before entering the study.

### Prediction scores

The description for the SOAR score has been published elsewhere [10, 11]. Briefly, the SOAR score is an 8-point scale. Variables include age (age≤65 years: 0 point; aged between 66 and 85 years: 1 point; age ≥85 years: 2 points.), stroke subtype (ischemic subtype: 0 point; hemorrhagic subtype:1 point, based on clinical and neuroimaging finding), oxford stroke classification project classification (partial anterior circulation or lacunar strokes: 0 point; posterior circulation: 1 point; total anterior circulation: 2 points, based on initial clinical assessment and neuroimaging), prestroke modified Rankin Scale (mRS, 0–2: 0 point; 3–4:1 point; 5: 2 points). The mSOAR score included all items of SOAR and added the baseline NIHSS at the time of first assessment on hospital arrival: 0 point, NIHSS score between 1 and 4; 1 point, NIHSS score between 5 and 10; 2 points, NIHSS score between 11 and 20; 2 points, NIHSS score ≥21 [12]. The sum of mSOAR and SOAR scores is the total points designated for these variables.

### Outcomes assessment

The outcome included discharge and 3-months mortality. The mortality included death from all causes.

### Statistical analysis

The continuous and categorical variables of patients’ baseline characteristics were presented as mean±SD or median (interquartile range, IQR) and percentages, respectively. Odds ratios (ORs) with 95% confidence intervals (95% CI) were calculated by using logistic regression analysis. The discriminatory power of the SOAR score and mSOAR score were assessed by the area under the receiver-operator curve (AUC) and 95% CI. An AUC statistic of 1.0 indicates perfect prediction, and of 0.5 indicates no better than random prediction. The *Z* test was used to compare the AUCs of different scores. Calibration was assessed by using the Pearson correlation coefficient test. The α level of significance was determined as *P*<0.05, 2-sided. All analyses were performed with SAS 9.3 software.

## Results

### Patient characteristics

A total of 12,415 ischemic stroke patients were enrolled in the CNSR registry who consented for follow-up. After excluding the cases with missing data (OCSP classification: n = 1195, baseline mRS: n = 173), 11073 patients were included in the present study (S1 Fig). The baseline characteristics of these patients were summarized in Table 1 and S1 Table. Among the 11073 patients, 38.1% subjects were female, and the average age was 65.5±12.3 years. The median NIHSS score was 5 (IQR 2–9). Among them, 60.2% had partial anterior circulation stroke (PACS), 12.0% had lacunar stroke (LACS), 18.0% had posterior circulation stroke (POCS) and 9.8% had total anterior circulation stroke (TACS), respectively.

**Table 1:** Characteristics of patients in included in the analysis (N = 11073).

Characteristics	
Female, n (%)	4217 (38.1)
Age, y (mean±SD)	65.5±12.3
NIHSS, median (IQR)	5.0 (2.0–9.0)
OCSP	
PACS	6670 (60.2)
LACS	1333 (12.0)
POCS	1990 (18.0)
TACS	1080 (9.8)
Pre-stroke disability	
mRS 0–2	10511 (95.0)
mRS 3–4	503 (4.5)
mRS 5	59 (0.5)
SOAR, median (IQR)	1.0 (0.0–1.0)
mSOAR, median (IQR)	1.0 (1.0–2.0)
**Discharge Mortality**	409 (3.7)
**3-Month Mortality**	885 (8.0)

NIHSS, National Institutes of Health Stroke Scale; OCSP, Oxfordshire Community Stroke Project classification; SOAR, stroke subtype, Oxfordshire Community Stroke Project classification, age, and prestrike modified Rankin; mSOAR, modified SOAR; mRS, modified Rankin Scale.

### The mSOAR/SOAR score and mortality

**Fig 1:**
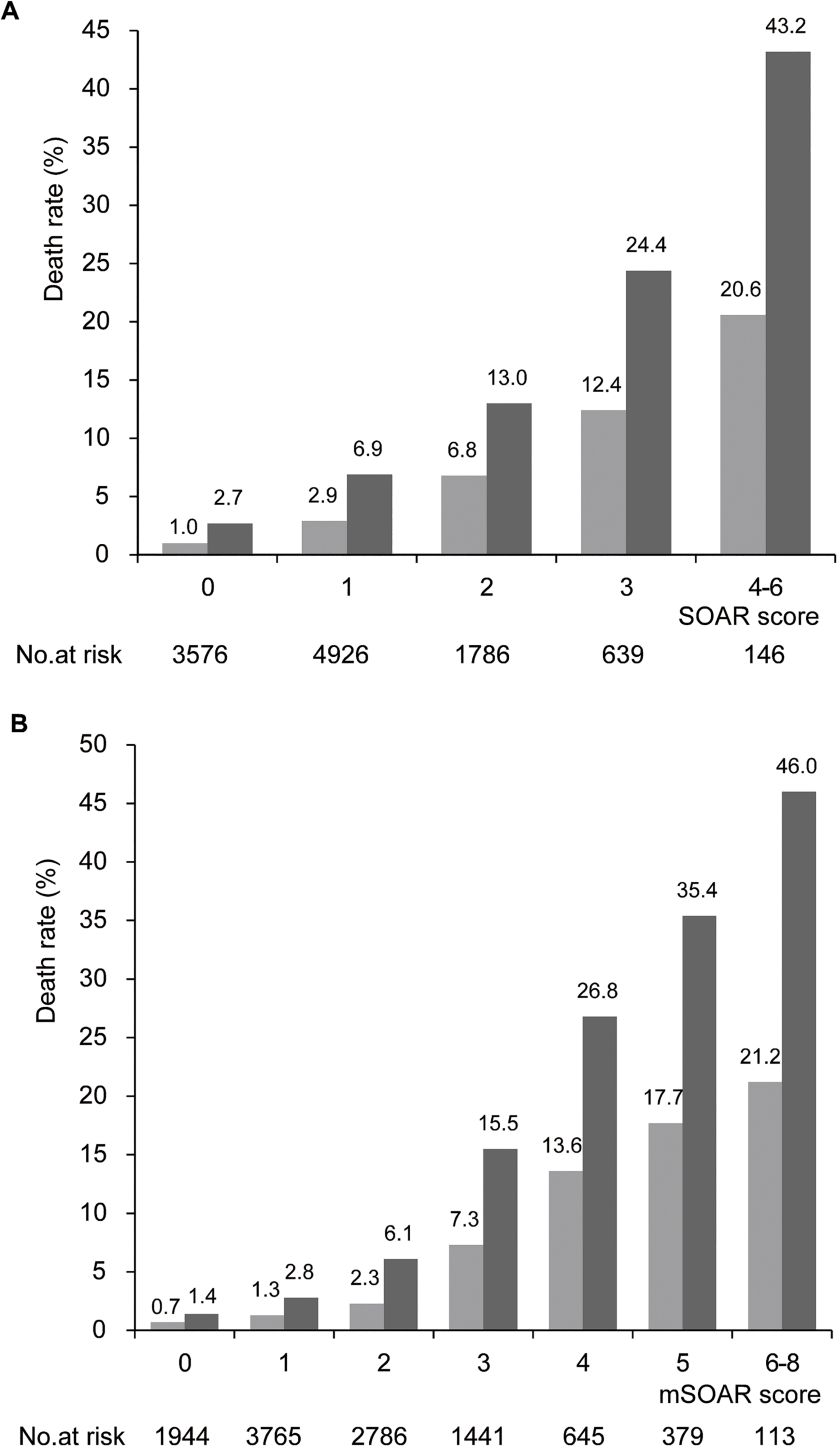
The discharge mortality and 3-month mortality were prognosticated by SOAR score (A) and mSOAR score (B). Patients with mSOAR score of 7 and 8 were merged into score of 6 for small sample size. Patients with SOAR score of 5 and 6 were merged into score of 4 for small sample size.

For patients with an mSOAR score of 0, 1, 2, 3, 4, 5 and 6 to 8 points, the discharge mortality were 0.7%, 1.3%, 2.3%, 7.3%, 13.6%, 17.7% and 21.2%, and the 3-month mortality were 1.4%, 2.8%, 6.1%, 15.5%, 26.8%, 35.4% and 46.0%, respectively (P_trend_<0.001, Fig 1B). For patients with SOAR score of 0, 1, 2, 3 and 4 to 6 points, the discharge mortality were 1.0%, 2.9%, 6.8%, 12.4% and 20.6%, and the 3-month mortality were 2.7%, 6.9%, 13.0%, 24.4% and 43.2%, respectively (P_trend_<0.001, Fig 1A). Moreover, as mSOAR and SOAR scores increased, the mortality showed a rising tendency (Table 2). Each 1 point increase in mSOAR score was associated with an OR of 1.98 for discharge mortality (95% CI, 1.86–2.11; *P*<0.001), an OR of 2.09 for 3-month mortality (95% CI, 1.99–2.19; *P*<0.001). Meanwhile, each 1 point increase in SOAR score was associated with an OR of 2.18 for discharge mortality (95% CI, 2.00–2.38; *P*<0.001), an OR of 2.17 for 3-month mortality (95% CI, 2.03–2.32; *P*<0.001).

**Table 2:** Predictive value of SOAR and mSOAR score for discharge and 3-month mortality.

	Outcome	Odds Ratio (95% CI)	AUC(95%CI)	P value
SOAR	Discharge Mortality	2.18 (2.00–2.38)	0.72 (0.70–0.75)	<0.001
	3-Month Mortality	2.17 (2.03–2.32)	0.70 (0.69–0.72)	<0.001
mSOAR	Discharge Mortality	1.98 (1.86–2.11)	0.78 (0.76–0.81)	<0.001
	3-Month Mortality	2.09 (1.99–2.19)	0.79 (0.77–0.80)	<0.001

CI, Confidence Interval; AUC, Area Under the Receiver-Operator Curves.

### ROC curve analysis comparing mSOAR score with other prediction scores

**Fig 2:**
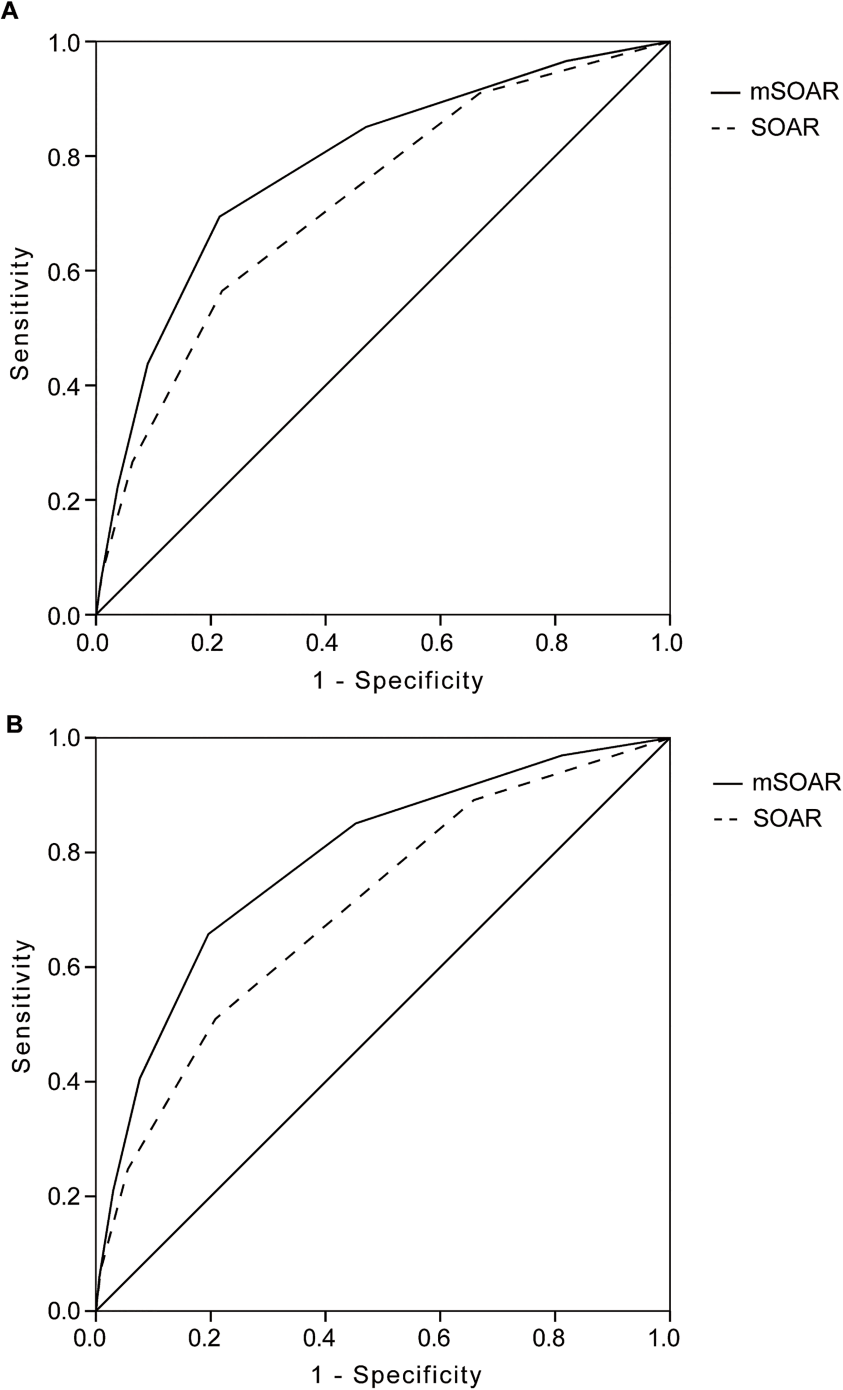
Receiver operating characteristic (ROC) curve analysis comparing mSOAR and SOAR score with other scores. The area under the receiver-operator curve (AUC) for prediction of discharges mortality (A) and 3-months mortality (B).

The AUC of ROC curve of mSOAR score was significantly greater than SOAR scores for predicting the mortality (Table 2; Fig 2). For predicting the discharge mortality, the AUC of mSOAR score was 0.78 (95% CI 0.76–0.81), compared with 0.72 for SOAR score (95% CI: 0.70–0.75) (*P* = 0.006). For predicting the 3-month mortality, the AUC of mSOAR score was 0.79 (95% CI: 0.77–0.80), compared with 0.70 for SOAR score (95% CI: 0.69–0.72) (*P*<0.001), respectively.

### Calibration ability of mSOAR and SOAR score

**Fig 3:**
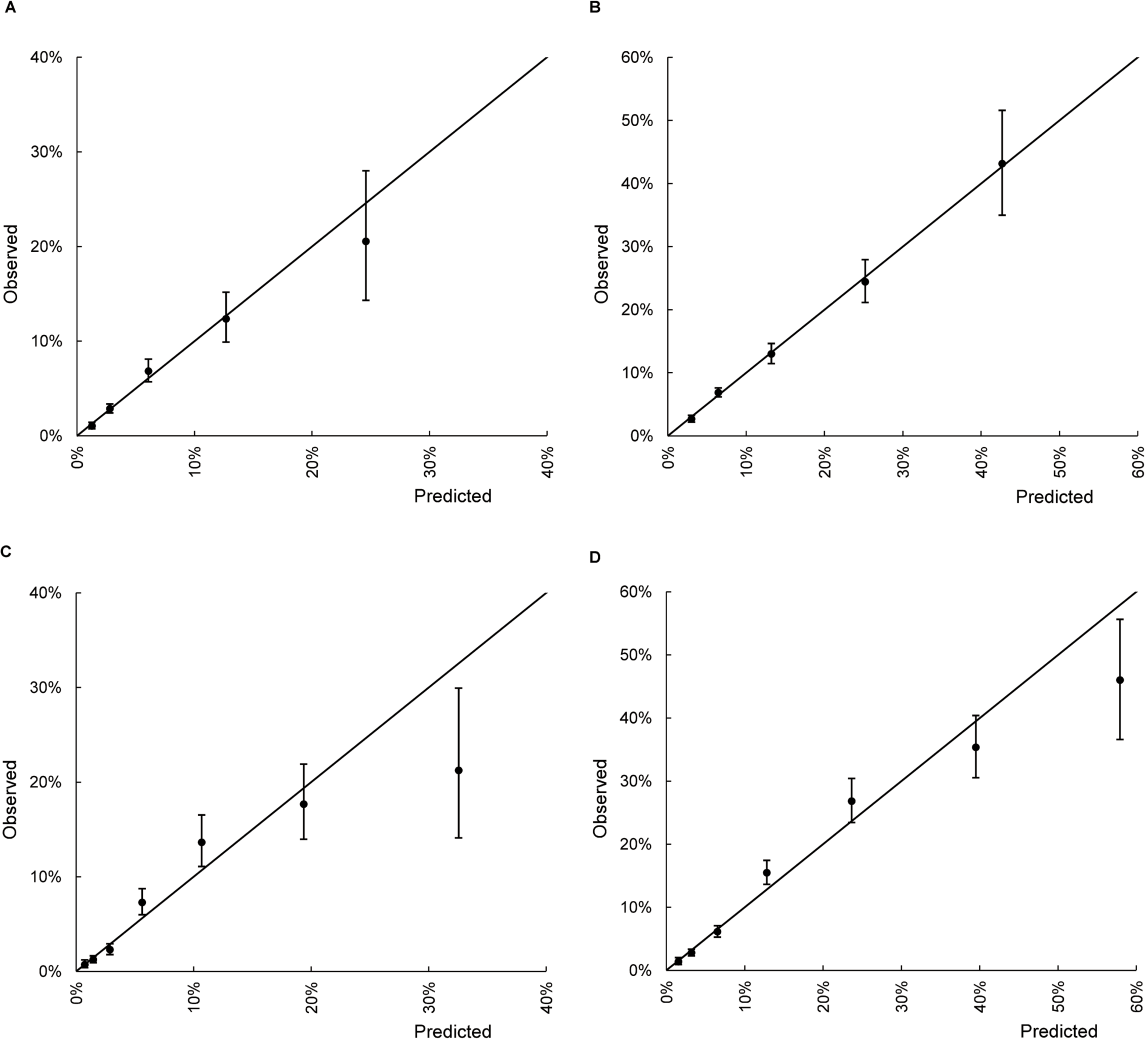
Calibration plot of (A) SOAR score for discharge mortality, (B) mSOAR score for discharge mortality, (C) SOAR score for 3-month mortality, and (D) mSOAR score for 3-month mortality. The vertical lines indicate the 95% confidence intervals of observed rates of mortality.

Calibration analysis of the SOAR score showed significant correlation between the predicted and observed probabilities of discharge mortality (*r* = 0.994, *P*<0.001) and 3-month mortality (*r* = 0.999, *P*<0.001). Moreover, the mSOAR score also showed significant correlation between the predicted and observed probabilities of discharge mortality (*r* = 0.945, *P* = 0.001) and 3-month mortality (*r* = 0.984, *P*<0.001) (Fig 3).

## Discussion

In this multicenter registry study of 11073 patients, the mSOAR and SOAR score showed a well prediction of discharge and 3-month mortality of acute stroke patients, especially by the mSOAR score.

Previous studies showed that the SOAR score was initially developed to predict 7-day mortality from a population of 12,355 British acute stroke patients (AUC: 0.79) [10]. It has validated to predict the 7-day mortality (AUC: 0.82) [11]. Meanwhile, the mSOAR score was modified by the original SOAR score, which was superior for predicting the early mortality of acute stroke patients (AUC: 0.83) [12]. Furthermore, these scores showed good performance for predicting the mortality in our Chinese acute stroke patient population, especially by the mSOAR score. The reason was likely that these scores contained some of the well-known and recognized factors for predicting the mortality and outcome of patients with acute stroke such as the age [15, 16]. OCSP classification [17, 18] and initial stroke severity evaluated by NIHSS score [19–21]. Especially in the OCSP classification, it included the clinical symptoms and neuroimaging. Different OCSP classification showed significant different prognosis of acute stroke patients with or without IV tPA [17, 18, 22]. Therefore, mSOAR score had performed better for predicting mortality than other scores.

The models for predicting acute stroke outcome were rapidly increased. However, the mSOAR and SOAR scores has their obvious superiority. First, the mSOAR and SOAR score was professionally developed to predict mortality. Second, this score contains only 5 easily obtainable variables, and it does not require laboratory testing. Thus it can be easily calculated.

Our study has several limitations. First, the study sites of CNSR were mainly localized at Chinese city regions, which have better stroke care quality than remote rural regions [14]. Second, follow-up mRS assessment was performed by telephone interviews, which may have biased this results. However, the telephone assessment of the mRS with a structured interview was shown a good agreement with face-to-face assessment [23]. Third, there were few patients with higher SOAR and mSOAR scores. Nearly 95% of patients had 0 to 2 points of pre-stroke mRS in CNSR cohort. Moreover, patients mainly had ischemic stroke, and they obtained 0 point for the “stroke type” item of mSOAR and SOAR score. This may have been the reason why the discharge mortality was 3.7% in this cohort, and why the mSOAR and SOAR socres were not high and the mSOAR score showed the good performance for predicting mortality in our study.

## Conclusions

Our study demonstrated that the mSOAR score has a good ability for predicting the discharge mortality and 3-month mortality in Chinese patients with acute stroke. The mSOAR score is a reliable and easy to use clinical instrument to predict mortality in acute stroke patients.

## Supporting information

S1 Fig Patient flow diagram. (TIF)

S1 Table Characteristics of patients in included in the analysis. (DOCX)

S1 File STROBE statement. Checklist of items that should be included in reports of observational studies.
(DOC)
