# Supplementary figures and images for: Validation of the mSOAR and SOAR scores to predict early mortality in Chinese acute stroke patients
Source: PLoS One. 2017 Jul 6;12(7):e0180444. doi: 10.1371/journal.pone.0180444 (PMC5500336; doi:10.1371/journal.pone.0180444)

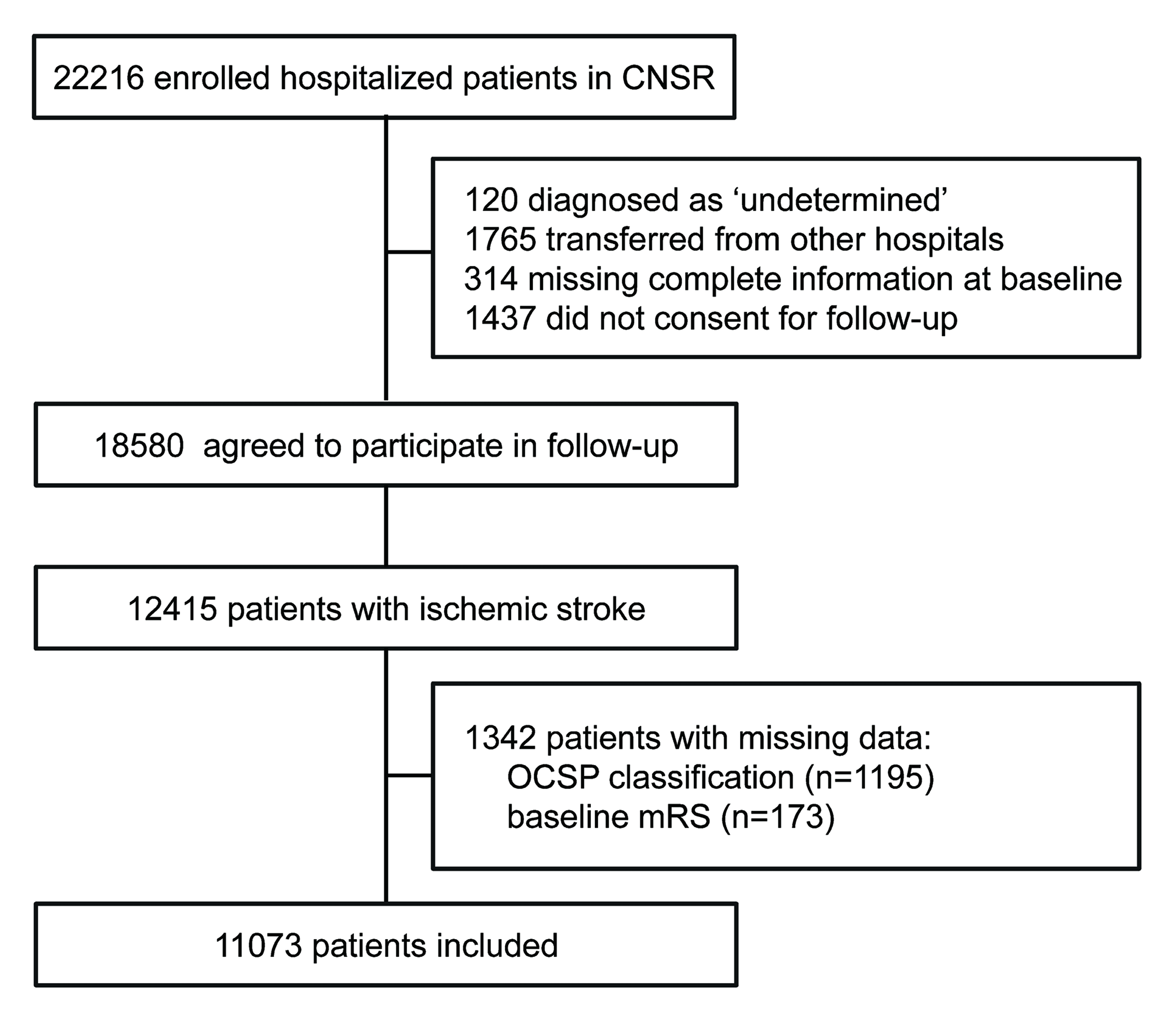

Supplement: S1 Fig — (TIF) [file pone.0180444.s001.tif]
