# Supplementary material for: Validation of the mSOAR and SOAR scores to predict early mortality in Chinese acute stroke patients
Source: PLoS One. 2017 Jul 6;12(7):e0180444. doi: 10.1371/journal.pone.0180444 (PMC5500336; doi:10.1371/journal.pone.0180444)
Supplement: S1 Table — (DOCX) [file pone.0180444.s002.docx]

**S1 Table.** Characteristics of patients in included in the analysis

| **Characteristics** | **Included (N=11073)** | **Excluded (N=1342)** | **P-value** |
| --- | --- | --- | --- |
| Female, n (%) | 4217 (38.1) | 540 (40.2) | 0.13 |
| Age, y (mean±SD) | 65.5±12.3 | 65.5±12.4 | 0.90 |
| Diabetes, n (%) | 2338 (21.1) | 303 (22.6) | 0.22 |
| Hypertension, n (%) | 6924 (62.5) | 873 (65.1) | 0.07 |
| Lipid disorder, n (%) | 1240 (11.2) | 150 (11.2) | 0.98 |
| Coronary Heart Disease, n (%) | 1593 (14.4) | 199 (14.8) | 0.66 |
| Stroke, n (%) | 3742 (33.8) | 492 (36.7) | 0.04 |
| Atrial Fibrillation, n (%) | 1154 (10.4) | 172 (12.8) | 0.01 |
